# Combination Strategies Involving Immune Checkpoint Inhibitors and Tyrosine Kinase or BRAF Inhibitors in Aggressive Thyroid Cancer

**DOI:** 10.3390/ijms23105731

**Published:** 2022-05-20

**Authors:** Francesca Ragusa, Silvia Martina Ferrari, Giusy Elia, Sabrina Rosaria Paparo, Eugenia Balestri, Chiara Botrini, Armando Patrizio, Valeria Mazzi, Giovanni Guglielmi, Rudy Foddis, Claudio Spinelli, Salvatore Ulisse, Alessandro Antonelli, Poupak Fallahi

**Affiliations:** 1 Department of Surgical, Medical and Molecular Pathology and Critical Area, University of Pisa, 56126 Pisa, Italy; francesca.ragusa@phd.unipi.it (F.R.); giusy.elia@phd.unipi.it (G.E.); sabrinapaparo@gmail.com (S.R.P.); balestri90@gmail.com (E.B.); chiara.botrini@gmail.com (C.B.); mazzivaleria@gmail.com (V.M.); claudio.spinelli@unipi.it (C.S.); 2 Department of Clinical and Experimental Medicine, University of Pisa, 56126 Pisa, Italy; silvia.ferrari@unipi.it; 3 Department of Emergency Medicine, Azienda Ospedaliero-Universitaria Pisana, 56124 Pisa, Italy; armandopatrizio125@gmail.com; 4 U.O. Medicina Preventiva Del Lavoro, Azienda Ospedaliero-Universitaria Pisana, 56124 Pisa, Italy; g.guglielmi@ao-pisa.toscana.it; 5 Department of Translational Research and New Technologies in Medicine and Surgery, University of Pisa, 56126 Pisa, Italy; rudy.foddis@med.unipi.it (R.F.); poupak.fallahi@unipi.it (P.F.); 6 Department of Surgical Sciences, ‘Sapienza’ University of Rome, 00161 Rome, Italy; salvatore.ulisse@uniroma1.it

**Keywords:** thyroid cancer, immunotherapy, new checkpoint inhibitors, tyrosine kinase inhibitors, PD-1 inhibitors, PD-L1 inhibitors

## Abstract

Thyroid cancer is the most common (~90%) type of endocrine-system tumor, accounting for 70% of the deaths from endocrine cancers. In the last years, the high-throughput genomics has been able to identify pathways/molecular targets involved in survival and tumor progression. Targeted therapy and immunotherapy individually have many limitations. Regarding the first one, although it greatly reduces the size of the cancer, clinical responses are generally transient and often lead to cancer relapse after initial treatment. For the second one, although it induces longer-lasting responses in cancer patients than targeted therapy, its response rate is lower. The individual limitations of these two different types of therapies can be overcome by combining them. Here, we discuss MAPK pathway inhibitors, i.e., BRAF and MEK inhibitors, combined with checkpoint inhibitors targeting PD-1, PD-L1, and CTLA-4. Several mutations make tumors resistant to treatments. Therefore, more studies are needed to investigate the patient’s individual tumor mutation burden in order to overcome the problem of resistance to therapy and to develop new combination therapies.

## 1. Introduction

Thyroid cancer (TC) is the most common (~90%) type of endocrine system tumor [1], accounting for 70% of the deaths from endocrine cancers [2]. Over the past two decades, its incidence has increased [3,4]. Among the risk factors, we find the following: female sex, history of goiter or thyroid nodules, family history of TC, radiation exposure, obesity, and low-iodine diet [5].

Although TC occurs more frequently in women than in men (three to four times more), it is more aggressive in men. In fact, men show more advanced disease and lower survival rates [6]. The main histologic types of TC are the following: (a) differentiated TC of follicular origin (DTC)—papillary (PTC, 80%), follicular (FTC, 11%), and Hürthle cells TC; (b) medullary TC (MTC, ~4%) (developed from C cells); and (c) anaplastic TC (ATC) (2% of all TCs) [7]. Poorly differentiated TC (PDTC) and ATC together represent about 5–10% of all TCs [8] and are responsible of most deaths [9,10,11].

Surgery is the principal therapeutical strategy in patients with DTC and MTC [12]. In DTC patients, after thyroidectomy, radioactive iodine (RAI) is used for the ablation of residual normal thyroid or residual metastatic tissue [13,14]. The subsequent follow-up is important to detect possible persistent/recurrent disease, and it includes neck ultrasound and basal/thyroid-stimulating hormone (TSH)-stimulated thyroglobulin (Tg) dosage, usually every 3–6 months during the first year, and then at different timings, which depend on the initial risk evaluation [15,16,17].

TCs range from indolent cancers, usually with low mortality, to very aggressive ones (for example, ATC) [18]. DTC represents more than 90% of TCs; in these cases, patients have a normal life expectancy [18]. In fact, only about 5% of DTC patients report metastases on lung, bone, or other sites at the diagnosis, and during the follow-up, ~15% of them show relapse in thyroid tissue or lymph nodes. In this case, the survival rate at 10 years decreases from 70% to 50% [19,20]. The progression of DTC causes the thyroid cells to lose the ability to capture iodide, thus becoming RAI-refractory. This affects the prognosis in a negative manner [19,20,21].

In the event of failure of RAI and TSH suppressive thyroid hormone (TH) treatment [22], metastatic DTC patients are treated with other therapeutic strategies, such as surgical resection, chemotherapy, and external beam radiotherapy (EBRT). However, they can lead to significant collateral adverse events (AEs) and actually have a palliative role, without prolonging survival whether used alone or in combination [23].

In recent years, new discoveries have been performed in the knowledge of the molecular/genetic basis of TC progression.

Most TCs are characterized by dysregulations involving the mitogen-activated protein kinase (MAPK) and phosphatidylinositol-3 kinase/mammalian target of rapamycin/protein-kinase B (PI3K/mTOR/Akt) signaling pathways that are crucial in the regulation of cellular proliferation [24,25].

MAPK hyperactivation is crucial in PTC initiation through point mutations of the BRAF oncogene. BRAF, a member of the RAF family of serine/threonine protein kinases downstream of RAS, is mutated with a higher prevalence in PTC (29–83%) [26,27,28,29]. PI3K/mTOR/Akt pathway activation has a central role in FTC development. Activating RAS mutations are found more frequently in FTC patients (28–68%), in up to 43% of follicular-variant PTCs (FVPTCs) [30], and in up to 47% of all non-invasive FVPTCs [31].

Additional mutations on other pathways, i.e., p53 and Wnt/β-catenin, have been described in case of TC progression and dedifferentiation to PDTC and ATC. Recent telomerase reverse transcriptase (TERT) promoter mutations have been reported in all histological subtypes of TC, with a significantly higher prevalence in aggressive and undifferentiated tumors, suggesting their role in TC progression [32].

New drugs, such as tyrosine kinase inhibitors (TKIs), have been described as emerging new therapies for progressive, aggressive, and refractory tumors. They are able to inhibit the oncogenic kinases (such as v-Raf murine sarcoma oncogene homolog B (^V600E^BRAF) and rearranged during transfection (RET)/PTC) or to inhibit the signaling kinases, such as Vascular Endothelial Growth Factor Receptor (VEGFR) or Platelet-Derived Growth Factor Receptor (PDGFR), that are implicated in the cell growth [33,34,35,36,37].

Most of these molecular targets might be new diagnostic/prognostic markers and could be targeted by new oncogenic therapies for TC [33,34,36].

In the last years, high-throughput genomics has been able to identify pathways/molecular targets involved in survival and tumor progression, revolutionizing personalized medicine. In the past, the therapies of TC were based on tumor type and histology; however, since several mutations make tumors resistant to conventional and already known treatments, recent approaches have focused on specific gene mutations and their dysregulation [38].

This inherent cancer heterogeneity contributes to the ever-increasing development of personalized and precision medicine. Personalized therapies may arrest tumor growth, as well as enhance anticancer immunity in a more efficient manner. The synergy between immunotherapies and targeted therapies leads to a personalized treatment. Targeted treatment acts on different oncogenic proteins involved in cancerogenesis, such as those of the MAPK pathways. Inhibitors against these pathways have led to promising results in several types of cancer, such as TC, melanoma, pancreatic cancer, Non-Small-Cell Lung Cancer (NSCLC), and colorectal cancer (CRC). A limitation of these targeted therapies is that, despite the considerable cancer size reduction, clinical responses are usually transient and often lead to cancer relapse after initial treatment. Although immunotherapy induces longer-lasting responses in cancer patients than targeted therapy—in particular, one form of immunotherapy, that is, the checkpoint blockade—its response rate is lower. The checkpoint blockade acts on checkpoint proteins involved in the suppression of the immune system. Individually targeted therapies and immunotherapies have many limitations, which can be overcome by their combination [39]. Indeed, recent evidence of this combination is represented by MAPK-targeted therapies, which can synergize with immune cells.

In this review, we discuss recent findings investigating combination strategies involving immune checkpoint inhibitors (ICIs) and TK or BRAF inhibitors in aggressive TC.

## 2. Methodology

The PUBMED database was searched by using the following search terms in the English language: thyroid cancer, immunotherapy, new checkpoint inhibitors, tyrosine kinase inhibitors, BRAF, MAPK, MEK, aggressive thyroid cancer, PD-1 inhibitors, PD-L1 inhibitors, and CTLA-4. The reference lists of selected articles were screened for additional relevant studies. Data from eligible studies were extracted and reviewed by the authors.

## 3. BRAF in TC

### 3.1. BRAF

The ^V600E^BRAF mutation, characterized by the substitution of glutamate with valine, is found in about 45% of PTCs (especially tall cell variants and classic papillary) and in 25% of ATCs [11,40]. This mutation, which causes the phosphorylation of a lot of targets (including mitogen activated protein kinase (MEK) and extracellular signal-regulated kinase (ERK)) by the activated BRAF kinase [41], is associated with malignant features, such as cancer aggressiveness and poor prognosis (larger tumoral size, metastases to lymph node, or distant) [42]. Regarding the prognostic role of the ^V600E^BRAF in PTC, there are studies showing contrasting results: (1) after adjustment for different risk factors (age, presence of lymph node or distant metastasis, and extra-thyroidal invasion), a large multicenter retrospective study showed no associated risk [43]; (2) a retrospective study on PTC patients showed different rates of cancer relapse in presence or absence of ^V600E^BRAF mutation (25% vs. 9.6%) [44].

### 3.2. Sorafenib in Aggressive DTC

Sorafenib (BAY 43–9006) is a multitarget TKI, able to inhibit several kinases, including RAF, RET, VEGFR-2 and -3, PDGFR, and KIT [45,46]. Sorafenib has been shown to exert anticancer effects both in preclinical models of tumor xenograft (such as breast cancer, colon, and non-small-cell lung) and in vitro by inhibiting TPC1 and TT growth, which carry the RET/PTC1 mutation and C634W RET, respectively [47]. It causes antiproliferative and antiangiogenic effects [48]. Its use has been approved by the FDA in the treatment of renal cell and hepatocellular carcinoma, as well as of metastatic DTC. Administration is performed orally (400 mg BID as maximum dosage), with a good patient tolerance.

Several studies have been conducted on sorafenib (Table 1).

An open-label phase II study enrolled 55 metastatic RAI-refractory TC patients (47% with PTC, 36% with FTC/Hürthle Cell, 9% with PDTC/ATC, and 8% with MTC). Progression-free survival (PFS) was better in PTC/FTC patients with the ^V600E^BRAF mutation than in the wild type (84 weeks vs. 54) [49].

A phase II trial investigated sorafenib (400 mg twice daily) to reinduce RAI uptake in 31 patients treated for 26 weeks. It reported partial response (PR) (25%), stable disease (SD) (34%), clinical response (59%), and PFS (58 weeks). No reinduction of RAI uptake was reported [50].

In another study, 13 patients with RAI-refractory PTC or FTC received sorafenib [41]. The remission rate was 20%, with a durable response rate in 66%, and a clinical benefit rate of 80%. Overall survival (OS) was 67% at 2 years, with a PFS of 19 months [51].

In the UPCC 03305 phase II study, 55 patients with advanced TC (85% with DTC/PDTC, 9% with ATC, and 6% with MTC) were enrolled. DTC/PDTC patients had a higher PFS with respect to the other TCs (96 vs. 93.6 weeks); in addition, 38% of them had a PR, and 47% a SD. DTC/PDTC patients (66%) had at least one mutation (in particular, 11% RET, 45% BRAF, 9% PIK3CA, and 19% RAS mutations), whereas 17% of patients had multiple mutations (60% in ATC) [52].

In another phase II trial, 15 patients with aggressive MTC and 19 with RAI-refractory DTC were administered with sorafenib (400 mg twice daily). The radiological response rate was 18%. A patient with a mutated BRAF exon 15 had an important response after 3 months [53].

In a phase II study, 31 RAI-refractory DTC patients received sorafenib (400 mg bid). The 31% of patients achieved a PR and 42% had a SD, after a median follow-up of 25 months. ^V600E^BRAF did not correlate with the progression of the disease [54].

In a phase II trial, 20 ATC patients were treated with sorafenib (400 mg bid), reaching a PR in 10%, and SD in 25%. Only 20% of patients reached a survival of 1-year, suggesting the ineffectiveness of sorafenib in ATC patients [55].

In the double-blinded randomized phase III (DECISION) trial, 417 patients with RAI-refractory, locally advanced, or metastatic DTC were enrolled, of which 207 received sorafenib and 210 received the placebo. PFS was higher in patients treated with the drug than in those treated with the placebo (10.8 vs. 5.8 months). PFS improved in all subgroups, with or without mutations. AEs occurred in 98.6% of sorafenib-treated patients [56].

Further studies have confirmed the results obtained with sorafenib in metastatic and progressive DTC [57,58]. 

Another phase II trial investigated the effect of sorafenib in patients with aggressive ATC or MTC, reporting the following: (a) in ATC, a median OS of 5.0 months and PFS of 2.8 months; and (b) in MTC, an objective RR of 25% and disease control rate in 75%. It was concluded that sorafenib was effective in MTC, but not in ATC [59].

In a phase II study, sorafenib was administered to 36 patients with metastatic TC (i.e., PTC, Hürthle cell, FTC, or ATC) (200 mg twice daily per os., combined with intravenous temsirolimus (25 mg weekly)), with a 22% PR, 58% SD, and 3% progressive disease (PD) [60].

A meta-analysis investigated the safety and efficacy of sorafenib in RAI-refractory DTC patients, showing that sorafenib improved PFS in comparison to the placebo [61].

A study compared sorafenib or sunitinib in 28 RAI-refractory metastatic DTC patients (26 treated with sorafenib as first-line therapy (eight patients switched successively to sunitinib), and two with sunitinib). The PR rate and mean PFS were 30.7% and 10.8 months, respectively, for sorafenib, and 37.5% and 6 months for sunitinib, as a second-line therapy [62].

### 3.3. Dabrafenib and Trametinib

Dabrafenib (GSK2118436) is a BRAF kinase inhibitor [63], whose antiproliferative effect has been shown in vitro in ^V600E^BRAF melanoma and in human colon tumor xenografts [69]. 

Trametinib (trade name Mekinist) is a MEK inhibitor drug with anticancer activity. It inhibits MEK1 and MEK2. Trametinib showed encouraging results for metastatic melanoma carrying the ^V600E^BRAF mutation in a phase III clinical trial.

In 2018, the combination of dabrafenib and trametinib was approved by the FDA for the treatment of locally advanced, unresectable, and metastatic ^V600E^BRAF-mutated ATC, with no possibility of loco-regional treatment.

Trametinib and dabrafenib activity was demonstrated by several studies (Table 1).

In a phase I study, 184 terminal patients with inoperable solid tumors (carrying the ^V600E^BRAF mutation) received dabrafenib (300 mg daily orally). A total of 14/184 patients had PTC, and nine of these survived for all the duration of the study, showing PR in 33% of cases [63]. However, as reported by many other clinical studies, BRAF-positive tumors exhibited resistance to dabrafenib in 6 to 7 months. To circumvent this problem, dabrafenib was used in combination with trametinib [70], a combination that was approved by the FDA in 2014 for the treatment of metastatic melanoma BRAF-V600E/K-positive [71].

Two patients with ^V600E^BRAF-positive ATC were administered with dabrafenib, and it was concluded that BRAF inhibitor monotherapy appears to obtain only temporary clinical improvement in ATC [64].

In a woman with an ATC with ^V600E^BRAF mutation (treated with EBRT, and then with pazopanib when the metastatic disease progressed to the neck and lung, with no benefits) the treatment with dabrafenib (150 mg bid) and trametinib (2 mg/day) led to a PR in 2 weeks. Then the disease progressed, and the patient died upon 6 months [65].

Moreover, another study reported a good clinical effect and a good tolerance in ATC patients with ^V600E^BRAF mutation, showing an overall response rate of 69% (95% CI, 41–89%), with seven ongoing responses [66].

Among 16 ATC patients (10 treated with lenvatinib, and 6 BRAF mutated patients with dabrafenib/trametinib), survival improved in the latter group of patients vs. those treated with lenvatinib [67].

Another study reported the efficacy and safety of dabrafenib plus trametinib in the ATC cohort of the phase II Rare Oncology Agnostic Research (ROAR) basket study. Thirty-six patients with ATC received dabrafenib (150 mg twice daily + trametinib 2 mg once) until disease progression, unacceptable toxicity, or death. The overall response rate was 56%, including three complete responses; the 12-month duration of response rate was 50%. The OS was 14.5 months, while the PFS was 6.7. The OS at 12 months was 43.2%, and at 24 months, it was 31.5% [68]. 

## 4. Immune Checkpoint Blockade

The goal of cancer immunotherapy is to improve the immune system ability to destroy cancer cells. The first protocol used involved the use of interleukin 2 (IL-2) for the treatment of metastatic melanoma [72]. IL-2 was able to enhance T-cell activation in a nonspecific way [73]. A high dosage of IL-2 is now not widely used as monotherapy, due to severe toxicity and low response rates [74]; on the contrary, IL-2 has been shown to be more effective when administered at a low dosage together with other treatments (e.g., adoptive cell transfer) [75].

Immune checkpoints, whose main role is to reduce the immune cell activation in order to keep the homeostasis of the immune system and inhibit the autoimmune process, represent an area for which new therapeutic strategies are emerging. Inside the tumor microenvironment, cancer cells upregulate the molecules of immune checkpoint in order to suppress the antitumor immune system response [76]. Specific monoclonal antibodies (mAbs) against immune checkpoints could be used as a strategy to invert the suppression of cancer-specific immune cells, such as T cells and natural killer (NK) cells [77,78]. Recently, Ribas et al. have demonstrated the efficacy of mAbs against immune checkpoint receptors, such as cytotoxic T-lymphocyte-associated protein 4 (CTLA-4), programmed cell death 1 (PD-1), and programmed cell death protein ligand (PD-L1) to treat multiple advanced cancers, such as NSCLC, melanoma, bladder, and head and neck cancers [79].

## 5. Immunotherapy and Targeted Therapy Combination in other Solid Cancers

Many cancers are able to evade cell death through the suppression of the immune system. One mechanism is the activation of the expression of PD-L1 by cytokines (e.g., IFN-gamma) released inside tumor-infiltrating lymphocytes (TILs). BRAF inhibitor-resistant melanoma cell lines, in which an increased expression of PD-L1 occurs, have shown the ability to evade the host’s immune system [80]. However, during the initial treatment stages, MAPK and BRAF inhibitors could boost the immune response against cancers, unless the patient develops resistance.

Intratumoral infiltration of T cells can be improved by BRAF and MEK inhibitors. Many studies have demonstrated an increase of T cells in BRAF-mutated melanomas after MAPK-pathway-inhibitors treatment, even if there is a loss of this increase with the progression of the therapy. CD8-effector T cells may be protected against death via stimulation of chronic T-cell receptor by MAPK-pathway inhibitors. The immune-stimulatory effects of MEK inhibitors include the increase of the expression of melanocyte-derived antigen, the increase of T-cell infiltration, and the reduction of the interaction among tumor cells and M2-like macrophages. Increased levels of cancer antigen could enhance antitumoral T-cell responses [80]. Nevertheless, MEK inhibitors can adversely affect the proliferation and viability of naive T cells and the secretion of IFN-gamma. 

MAPK inhibitors can stimulate transient responses. It was demonstrated that melanoma patients with BRAF mutation show some short-term advantage after targeted treatments (e.g., MAPK inhibitors). On the contrary, it was suggested that immunotherapies such as immune checkpoint blockades are able to stimulate longer-term responses in about 1/3 of patients [81]. In addition, it was shown that tumor immune infiltration and control could be improved by short-term blockage of MEK and BRAF, together with anti-PD-1/L1 Abs, in a CD8-T cells-dependent way. Monotherapies-related limitations could be bypassed through the combination of MAPK inhibitors and ICIs.

The combination of ICIs (especially anti-PD-1/L1) with MEK and/or BRAF inhibitors are now under evaluation through several clinical trials. The toxicity of each single agent, if used as combined therapy, can only be evaluated with long-term studies. Atkins et al. conducted a phase I study on melanoma, both BRAF-mutated melanoma and BRAF wild type, which showed a tolerable safety profile for an anti-PD-L1 antibody when used together with trametinib and dabrafenib [82]. A phase II study on advanced melanoma patients (NCT02625337) investigated the combination of pembrolizumab with dabrafenib and trametinib, given intermittently or continuously [83,84].

Optimizing the sequence in which ICIs and targeted therapies are given could have potential benefits, as it could reduce the toxicity and costs related to simultaneous treatment strategies used for long periods. Targeted therapies/immunotherapies integration can be useful to overcome the limitations of the single therapeutic approach and to potentiate the response to monotherapy.

Here, we discuss clinical trials conducted in patients with not TC cancers, such as melanoma, NSCLC, and CRC (Table 2).

### 5.1. Melanoma

Surgery is the primary therapeutic strategy used to treat patients with early stage melanoma, but not in the case of advanced melanoma (because of the high rate of metastasis). Patients with late-stage melanoma are usually treated with conventional chemotherapy, even if the response rate is very poor (~5%) [92,93]. Recently, the strategy of combination therapy (targeted therapies plus immunotherapies) allowed to improve the prognosis of melanoma.

More than 50% of patients with melanoma carry the ^V600^BRAF mutation, which causes constitutive activation of the MAPK signaling [94]. In 2011 the FDA approved the use of vemurafenib, a BRAF inhibitor, for patients with advanced melanoma, as a result of which rates of OS and PFS improved significantly [95]. In 2013, the FDA approved dabrafenib as another BRAF inhibitor; it has an efficacy similar to vemurafenib but with a lower frequency of side effects. Other drugs combination, such as dabrafenib plus trametinib, are expected to be associated with a higher response rate than dabrafenib monotherapy [96]. However, these drugs allow to obtain remarkable clinical responses in the short-term, but only rarely in long-term, due to the acquired resistance [97].

In addition to BRAF inhibitors, MEK inhibitors have been developed, including trametinib. Trametinib was the first FDA-approved MEK inhibitor for the treatment of advanced melanoma. In particular, it inhibits MEK1/2 [98]. For this kind of tumor, recently the FDA also approved the combination of encorafenib (a BRAF inhibitor) and binimetinib (a MEK inhibitor), and this combination is able to improve PFS and OS in patients with BRAF-mutated melanoma [99]. A limitation of these treatments is that, after several months of treatment, the rate of clinical responses tends to decrease [100].

Several clinical trials have investigated the combination of targeted therapies and immunotherapies, specifically ICIs, in order to improve the clinical response (Table 2) [83].

### 5.2. NSCLC

Lung cancer has a high frequency and is related to mortality regardless of gender. Early stage (stages I, II, and IIIA) NSCLC is usually treated with surgical resection [101], but in 80% of cases, lung cancers are diagnosed when they are already in an advanced stage, and surgery is no longer indicated. Furthermore, tumor relapse may occur a few years after surgical resection [102]. In patients with advanced NSCLC, chemotherapy is, thus, the first-line therapy used [101].

For the treatment of NSCLC patients, immunotherapies have been developed. Several clinical trials have demonstrated the efficacy of anti-PD-1/PD-L1 antibodies, with significant responses and low toxicities [103]. Chemotherapy, which increases the expression of PD-L1 on tumor cells and the number of TILs, has been demonstrated to have few effects in patients characterized by elevated levels of PD-L1 and low levels of causative mutations [104]; thus, the combination between ICIs and chemotherapy could have promising clinical results [105,106].

In recent decades, therapeutic strategies targeting epidermal growth factor receptor (EGFR) and anaplastic lymphoma kinase (ALK) mutations have made it possible to achieve substantial advances in the treatment of NSCLC patients [107]. Most EGFR mutations, including exon 21 L858R and exon 19 deletions mutations, cause the constitutive activation of downstream pathways, such as MAPK, signal transducer and activator of transcription (STAT), and PI3K [108].

Several clinical trials investigated the combining of MAPK inhibitors and ICIs (Table 2) [83].

### 5.3. CRC

CRC is the third most common cancer in the world [109]. The OS of metastatic CRC patients is low. Surgery and subsequent adjuvant chemotherapy represent the standard treatment [110,111]. Mutations on BRAF, KRAS, SMAD4, and p53 genes represent the most common mutations, which have an important relevance in the development of CRC metastasis [110].

ICIs which have shown to be effective in many types of cancer (anti-PD-1 mAb (pembrolizumab and nivolumab), anti-PD-L1 Ab (avelumab, durvalumab, and atezolizumab), and anti-CTLA-4 Ab (tremelimumab and ipilimumab)), are not equally effective in treating CRC [111]. Nevertheless, if they are used in combination with MAPK-pathway-targeted therapy, they result in being more efficacious, as demonstrated by several clinical trials (Table 2) [83].

## 6. Immunotherapy in DTC

As the tumor microenvironment is the primary scenario involved in tumor progression and treatment response, new therapeutic strategies target the TC immune panorama. Immune checkpoints, such as PD-1 and its ligand PD-L1, and CTLA-4 inhibitors work by altering the interaction immune system/cancer cells [112]. Several studies have investigated the PD-1/PD-L1 expression in TC for prognostic and diagnostic purposes [113]. Aghajani et al. have demonstrated the association of PD-L1 in TC with tumor relapse and poor survival [114]. According to the TCGA database analyses, lymph node metastasis, extrathyroid invasion, and shorter disease-specific survival are linked to increased PD-L1 mRNA expression [115], as supported by two studies [116,117]. However, since DTC has few mutations, it is poorly immunogenic. Indeed, it has been demonstrated that TC responds poorly to immunotherapy with checkpoint inhibitors [118,119]. The efficacy of immunotherapy could be improved by combined treatments, as evaluated in several clinical studies conducted on solid tumors, such as TC [56,66,116,118,120,121,122,123,124,125,126,127,128,129,130,131,132,133,134,135,136,137,138,139,140,141,142,143,144,145,146,147,148,149].

## 7. Immunotherapy and Targeted Therapy in Aggressive TC

ATC is the rarest TC, but the most aggressive one, causing ~50% of the deaths due to TCs. Median survival from the diagnosis is usually about 6 months, because of its aggressiveness and the absence of an efficacious therapy [150].

The standard treatment of ATC includes surgical debulking, accelerated hyperfractionated EBRT, and chemotherapy, which permit patients to reach about 10 months of median survival. It is still challenging to predict the ATC patient clinical therapy responsiveness [150].

ATC shows a high frequency of ^V600E^BRAF mutations, and it is associated with an immunosuppressive microenvironment [151].

Combination therapy is particularly effective in de-differentiated tumors, such as widely invasive Hürthle cell TC and ATC, in which there are a large number of mutations that introduce immunogenicity [152,153], as suggested by some studies described below.

### 7.1. PLX4720 

Gunda et al. have assessed the combined treatment of PLX4720 (^V600E^BRAF inhibitor) and an anti-PD-1/PD-L1 Ab in an immunocompetent murine model of orthotopic ATC. They conducted an immune profiling of myeloid and lymphoid lineage cells at the moment of the maximum response to treatment and tumor regrowth. They demonstrated that the combined treatment results in a significant increase in the mouse survival. At the moment of maximal cancer reduction, a decrease of Ki67 proliferative index, an increase of tumor CD8+ cytotoxic T cells, FoxP3+ Tregs, and NK cells, as well as an increase of granzyme B staining and IFN-gamma production, occur, confirming the increasing of cytotoxicity. However, no complete responses were observed, and tumor regrowth emerged after 2–3 weeks of combination therapy, rapidly causing the mouse to die. Cancer regrowth was associated with decreases in CD8 + T cells and NK cells and loss of granzyme B and IFN-gamma production, confirming the attenuation of inflammation. At the time of maximum tumor shrinkage, there was an increased number and cytotoxicity of CD8 + T cells and NK cells, an increased number of M1 polarized tumor-related macrophages (TAMs) and a decreased number of myeloid-derived suppressor-like cells. On the contrary, at the time of tumor regrowth, there was a decrease in TAMs and an increase in M2 polarization. Hence, the combined treatment drastically decreased tumor volume, prolonging survival and improving the anticancer immune profile in a mouse ATC model, but tumor regrowth was inevitable [151].

### 7.2. Pembrolizumab 

Kinase inhibitors (KIs) have demonstrated a good effectiveness in the ATC treatment; nevertheless, these cancers eventually acquire KI resistance, and patients succumb to their own disease.

KIs targeting BRAF, MEK, and VEGFR have shown promise in managing ATC in a clinical study and in the real world [66,67,116,154]. Although these therapies manage to improve the median OS of ATC patients, the tumors eventually show resistance, with subsequent disease progression and death [12,42]. It is therefore a priority to identify rescue treatments for cancers that progress despite KI treatment.

ATC tumors express the PD-L1 on the tumor surface; there is a diffuse infiltration of the tumor with T-lymphocytes bearing the PD-1 receptor. Pembrolizumab, a mAb against the PD-1 receptor, represents a safe and effective salvage therapy to be added to KI therapy at the time of progression, since the immune microenvironment could be less permissive at the time of progression on KI therapy [155].

Cabanillas et al. reported the case of an unresectable/end-stage/locally advanced ATC patient who was treated at first with trametinib and dabrafenib, and subsequently with pembrolizumab. A PR was reached, allowing surgical resection and chemoradiation [156].

Iyer et al. have explored the effectiveness of pembrolizumab when added to KIs at the moment of ATC progression in patients of MD Anderson Cancer Center between August 2016 and August 2017. BOR was evaluated through the RECIST v1.1 criteria: 42% of patients (5/12) with PR, 33% (4/12) with SD, and 25% (3/12) with PD. Then it was assessed the PFS from the beginning of pembrolizumab, as well as median OS from the beginning of KIs, and from the addition of pembrolizumab: from the start of KIs, the median OS was 10.43 months; after the addition of pembrolizumab, the PFS and median OS were 2.96 months and 6.93 months, respectively. These results suggest that pembrolizumab may represent an effective rescue treatment to add to KIs as cancers progress. Furthermore, the addition of pembrolizumab could occur at any time during KI treatment in order to maximize benefit from the combination treatment. Nevertheless, better therapeutic strategies that include immunotherapy should be investigated for the treatment of patients with ATC [155].

A recent study described six consecutive BRAF-mutated ATC patients with loco-regional advanced disease who received dabrafenib and trametinib, surgical treatment, and adjuvant chemoradiation. Among them, three were also administered with pembrolizumab. At 6 months, the OS was 100%, and it was 83% at 1 year [157].

Sukari et al. [158] recently reported two cases of ATC in order to underline the importance of histopathological diagnosis of tissues, the role of molecular tests, and the potential role of ICIs:

— Case 1: A 49-year-old man with ATC (diagnosed after a re-review of the histological examination) was treated with concurrent chemotherapy and radiation therapy after surgical resection. A post-treatment PET-CT scan did not highlight residual FDG uptake. The patient was monitored for 14 months, when he suddenly experienced pain in his left arm that later turned out to be a humerus metastasis with a pathological fracture. The histological investigation of metastasis during surgical fixation detected the presence of poorly differentiated malignant cells in line with ATC, so the patient was treated with pembrolizumab (three cycles). Nevertheless, diffuse bone metastases and a new liver injury were later individuated.
— Case 2: A 61-year-old woman was diagnosed with ATC with extrathyroid expansion and metastasis in lymph nodes and lungs. She was treated with concurrent chemotherapy plus radiation therapy. A post-treatment PET-CT scan highlighted residual FDG uptake in the pulmonary nodules. Lenvatinib was then initiated until the ^V600E^BRAF mutation was identified in the tumor. After that, she was treated with dabrafenib 150 mg PO BID and trametinib 2 mg PO daily. A reduction of the size of the left lung lesion was observed at nine months. Two months later, for the appearance of the metastasis in the right lung, the patient initiated carboplatin, paclitaxel, and pembrolizumab; after four cycles of this therapy, she continued dabrafenib plus trametinib, showing a stability of the disease for 10 months.

Sukari et al. have finally affirmed that ATC immunotherapy with single agent could not be as efficacious (case 1) as in combination (case 2), while other case reports have reported the success of ICIs as single agent [158,159].

Kulkarni et al. reported a case of a 71-year-old ATC man with aggressive metastasis of the lung and ^V600E^BRAF mutation, who initiated dabrafenib 150 mg PO (twice) and trametinib 2 mg PO. After 6 weeks, he presented with fever, and, for this reason, the therapy was stopped and restarted 10 days after with reduced dose of dabrafenib 100 mg PO BID and trametinib 1.5 mg PO that were continued for 6 weeks until the appearance of side effects (recurrent fevers, hearing loss, and uveitis). Since his tumor had a PD-L1 expression of 90%, pembrolizumab was given, but after five cycles, a recurrence of ATC was observed in the right lung and mediastinal lymph nodes [160].

### 7.3. Spartalizumab

Capdevila et al. conducted a phase I/II study on patients with ATC who were treated with spartalizumab. They enrolled 42 patients with locally advanced and/or metastatic ATC. Patients received 400 mg of spartalizumab intravenously once every 4 weeks. The overall RR was obtained according to RECIST v1.1 criteria.

The overall RR was 19%, with a complete response in three patients and a PR in 5 patients. The RR were higher in PD-L1–positive (8/28; 29%) vs. PD-L1–negative (0/12; 0%) patients. Responses were observed in patients with or without BRAF mutation, with a 1-year survival of 52.1% in patients PD-L1–positive.

Side effects of spartalizumab were similar to those of other PD-1-targeting mAbs [161,162,163].

This was the first clinical trial to show the responsiveness of ATC to PD-1 blockade [164].

### 7.4. Oncolytic Herpes Simplex Virus 

About 30–60% of PDTCs and ATCs show mutations in the BRAF gene; however, specific inhibitors that target oncogenic BRAF have shown a short-lasting therapeutical benefit as single agents. This underlines the necessity for improved treatment strategies, such as new combinations.

Using a ^V600E^BRAF-driven mouse model of ATC, Crespo-Rodriguez et al. assessed the therapeutic effectiveness of combining BRAF inhibition/oncolytic herpes simplex virus (oHSV). 

Samples from tumor-bearing mice were analyzed in order to immunologically characterize the effects of various treatments.

The authors characterized the immune landscape in vivo after treatment with BRAF inhibitor and found only few immune modifications. For this reason, they added oncolytic virotherapy to BRAF inhibition in TC to obtain a more favorable tumor immune microenvironment, as well as increase the inflammatory status of cancers and ameliorate BRAF inhibitor therapy. In advance, they demonstrated that TC cells were susceptible to oHSV infection and that this condition was related to the activation of the immune cancer microenvironment in vivo. Subsequently, they demonstrated enhanced therapeutic responses following the combination BRAF inhibition/oHSV in vivo, even if without synergistic effects in vitro, and confirmed that the dominant effect of oHSV in this setting was immune-mediated.

The detected gene and protein expression data increased T-cell and NK-cell activation in the cancer after combined treatment. However, the success of this association was nullified after T cells or NK cells were depleted in vivo. Moreover, an upregulation of PD-L1 and CTLA-4 after combined treatment and an improvement of combination therapy by the blockade of the PD-1/PD-L1 axis or CTLA-4 have been demonstrated [165].

## 8. Conclusions

TC is the most common type of endocrine-system tumor [1], accounting for 70% of endocrine-cancer deaths [2].

Recent discoveries have been performed in the knowledge of the molecular/genetic basis of TC progression. New drugs, such as TKIs, have been described as emerging new therapies for progressive, aggressive, and refractory tumors, since they are able to block the oncogenic kinases or to block the signaling kinases associated with cell growth [33,34,35,36].

The high-throughput genomics have recently identified pathways/molecular targets involved in survival and tumor progression.

Separately, targeted therapies and immunotherapies have many limitations. Clinical responses to targeted therapy are generally transient and often lead to cancer relapse after initial treatment; and immunotherapy has a low response rate. 

These individual limitations can be overcome by combining them. Indeed, combination therapies target signal transduction cascades required for tumor cell survival and maintenance.

Since a lot of different mutations make tumors resistant to treatments, knowing that the patient’s individual tumor mutation burden is important in selecting the optimal personalized combination regimen. This can be an effective strategy to overcome the problem of resistance to therapy and to develop new combination therapies.

## Figures and Tables

**Table 1:** In vivo studies on TC treated with different KIs.

Study	Indication	Main Results	Reference
An open-label phase II study enrolled 55 metastatic RAI-refractory TC patients (47% with PTC, 36% with FTC/Hürthle Cell, 9% with PDTC/ATC, and 8% with MTC) treated with sorafenib (400 mg orally bid).	Metastatic RAI-refractory TC	PFS was better in PTC/FTC patients with ^V600E^BRAF mutation than in wild type (84 weeks vs. 54).	[49]
A phase II trial investigated sorafenib (400 mg twice daily) to reinduce RAI uptake in 31 patients treated for 26 weeks.	RAI-refractory DTC	The study reported partial response (PR) (25%), stable disease (SD) (34%), clinical response (59%), and progression-free survival (PFS) (58 weeks). No reinduction of RAI uptake was reported.	[50]
In a study, 13 patients with RAI-refractory PTC or FTC received sorafenib.	RAI-refractory PTC or FTC	The remission rate was 20%, with a durable response rate in 66% and a clinical benefit rate of 80%. Overall survival (OS) was 67% at 2 years with a PFS of 19 months.	[51]
In a UPCC 03305 phase II study, 55 patients with advanced TC (85% with DTC/PDTC, 9% with ATC, and 6% with MTC) were enrolled and treated with sorafenib (400 mg bid).	Advanced TC (85% with DTC/PDTC, 9% with ATC, and 6% with MTC)	DTC/PDTC patients had a higher PFS with respect to the other TCs (96 vs. 93.6 weeks); in addition, 38% of them had a PR, and 47% a SD.	[52]
In a phase II trial, 15 patients with aggressive MTC and 19 with RAI-refractory DTC were administered with sorafenib (400 mg twice daily).	Aggressive MTC and RAI-refractory DTC	The radiological response rate was 18%. A patient with a mutated BRAF exon 15 had an important response after 3 months.	[53]
In a phase II study, 31 RAI-refractory DTC patients received sorafenib (400 mg bid).	RAI-refractory DTC	The 31% of patients achieved a PR and 42% had a SD, after a median follow-up of 25 months. ^V600E^BRAF did not correlate with the progression of the disease.	[54]
In a phase II trial, 20 ATC patients were treated with sorafenib (400 mg bid).	ATC	Patients reached a PR in 10% and SD in 25%. Only 20% of patients reached a survival of 1-year, suggesting the ineffectiveness of sorafenib in ATC patients.	[55]
In a double-blinded randomized phase III (DECISION) trial, 417 patients with RAI-refractory, locally advanced or metastatic DTC were enrolled, of which 207 received sorafenib and 210 received a placebo.	RAI-refractory, locally advanced or metastatic DTC	PFS was higher in patients treated with the drug than in those treated with placebo (10.8 vs. 5.8 months). PFS improved in all subgroups, with or without mutations. AEs occurred in 98.6% of sorafenib-treated patients.	[56]
Outside of clinical trials, 62 patients were treated with sorafenib (62%), sunitinib (22%), and vandetanib (16%).	PTC, FTC, Hürthle cell, PDTC, MTC	Among the 39 sorafenib and 12 sunitinib treatments in DTC patients, partial response rate was 15 and 8% respectively. In the 11 MTC patients treated with vandetanib, 36% had PR. Median PFS was similar in second-line therapy compared with first-line sorafenib or sunitinib therapy (6.7 vs. 7.0 months) in DTC patients, but there was no PR with second- and third-line treatments. Bone and pleural lesions were the most refractory sites to treatment.	[57]
Off-label observational study. Sorafenib 400 mg twice daily was evaluated. Therapy duration was 12 ± 3 months (range 6–16 months).	Progressive radioiodine resistant metastatic TC	One patient showed a PR with tumor regression of −35% six months after the beginning of the treatment; five patients exhibited SD, and two patients had progressive disease (PD) and died.	[58]
A phase II trial investigated the effect of sorafenib in patients with aggressive ATC or MTC.	Aggressive ATC or MTC	The study reported, (a) in ATC, a median OS of 5.0 months and PFS of 2.8 months; and (b) in MTC, an objective response rate of 25% and disease control rate in 75%. It was concluded that sorafenib was effective in MTC, but not in ATC.	[59]
In a phase II study, sorafenib was administered to 36 patients with metastatic TC (i.e., PTC, Hürthle cell, FTC, or ATC) (200 mg twice daily per os., combined with intravenous temsirolimus (25 mg weekly)).	Metastatic TC (i.e., PTC, Hürthle cell, FTC, or ATC)	The study reported: 22% PR, 58% SD, and 3% PD.	[60]
A meta-analysis investigated the safety and efficacy of sorafenib in RAI-refractory DTC patients.	RAI-refractory DTC	Sorafenib improved PFS in comparison to placebo.	[61]
A study compared sorafenib or sunitinib in 28 RAI-refractory metastatic DTC patients (26 treated with sorafenib as first-line therapy (8 patients switched successively to sunitinib), and 2 with sunitinib).	RAI-refractory metastatic DTC	PR rate and mean PFS were 30.7% and 10.8 months, respectively, for sorafenib, and 37.5% and 6 months for sunitinib, as a second-line therapy.	[62]
In a phase I study, 184 terminal patients with inoperable solid tumors (carrying the ^V600E^BRAF mutation) received dabrafenib (300 mg daily orally).	PTC	A total of 14/184 patients had PTC, and nine of these survived for all the duration of the study, showing PR in 33% of cases. However, as reported by many other clinical studies, BRAF-positive tumors exhibited resistance to dabrafenib in 6 to 7 months.	[63]
In a case report, two patients with ^V600E^BRAF-positive ATC were administered with dabrafenib.	^V600E^BRAF ATC	BRAF inhibitor monotherapy appears to obtain only temporary clinical improvement in ATC.	[64]
A woman with an ATC with ^V600E^BRAF mutation (treated with EBRT, and then with pazopanib when the metastatic disease progressed to the neck and lung, with no benefits) was treated with dabrafenib (150 mg bid) and trametinib (2 mg/day).	^V600E^BRAF ATC	The treatment led to a PR in 2 weeks. Then the disease progressed, and the patient died upon 6 months.	[65]
In a phase II open-label trial, patients with BRAF V600E-mutated ATC received dabrafenib 150 mg twice daily and trametinib 2 mg once daily until unacceptable toxicity, disease progression, or death.	^V600E^BRAF ATC	Good clinical effect and a good tolerance; overall response rate of 69% (95% CI, 41–89%), with 7 ongoing responses.	[66]
Real-World Experience: ten patients (eight BRAF wild type and two ^V600E^BRAF mutant tumors) were started on lenvatinib, and six with ^V600E^BRAF-mutated tumors received a combination of dabrafenib plus trametinib.	ATC	In the entire cohort, 6/16 (38%) had a PR, 6/16 (38%) had SD, and 2/16 (12%) had PD. Median follow-up time was 11.8 months. Median progression-free survival was 3.7 months (CI 1.8–7.6) in the entire cohort, 2.7 months for lenvatinib, and 5.2 months for dabrafenib plus trametinib. Median OS was 6.3 months (CI 1.8–7.6) for the entire cohort, 3.9 months for lenvatinib, and 9.3 months for dabrafenib plus trametinib.	[67]
In a phase II Rare Oncology Agnostic Research (ROAR) basket study, thirty-six patients with ATC received dabrafenib (150 mg twice daily + trametinib 2 mg once) until disease progression, unacceptable toxicity, or death.	^V600E^BRAF ATC	The overall RR was 56%, including 3 complete responses; the 12-month duration of response rate was 50%. OS was 14.5 months, while PFS was 6.7. The OS at 12 months was 43.2%, and at 24 months, it was 31.5%.	[68]

**Table 2:** Clinical trials conducted in patients with melanoma, NSCLC, and CRC.

National Clinical Trial (NCT) Number	Indication	Drugs	Phase	Reference
NCT01400451	Melanoma	Vemurafenib and ipilimumab	I	The study is terminated [83,85,86,87]
NCT01673854	Melanoma	Vemurafenib, followed by ipilimumab	II	The study is completed [83,88]
NCT03235245	Melanoma	Ipilimumab and nivolumab preceded or not by a targeted therapy with encorafenib and binimetinib	II	The study is in recruiting phase [83]
NCT02967692	Melanoma	Spartalizumab/trametinib/dabrafenib	III	The study is active, not recruiting [83,89,90]
NCT02902042	Melanoma	Encorafenib/binimetinib/pembrolizumab	I/II	The study is completed [83,91]
NCT02858921	Melanoma	Trametinib, dabrafenib and/or pembrolizumab, administered before surgery	II	The study is active, not recruiting [83]
NCT03991819	NSCLC	Binimetinib and pembrolizumab	I/Ib	The study is in recruiting phase [83]
NCT03600701	NSCLC	Atezolizumab and cobimetinib	II	The study is in recruiting phase [83]
NCT03581487	NSCLC	Selumetinib, durvalumab, and tremelimumab	I/II	The study is in recruiting phase [83]
NCT03299088	NSCLC	Pembrolizumab and trametinib	Ib	The study is active, not recruiting [83]
NCT03225664	NSCLC	Trametinib and pembrolizumab	Ib/II	The study is active, not recruiting [83]
NCT04044430	CRC	Binimetinib, encorafenib, and nivolumab	I/II	The study is active, not recruiting [83]
NCT03428126	CRC	Durvalumab and trametinib	II	The study is active, not recruiting [83]
NCT03374254	CRC	Pembrolizumab and binimetinib, with respect to the combination of chemotherapy and pembrolizumab, with/without binimetinib	Ib	The study is active, not recruiting [83]
